# Uncovering the neural control of laryngeal activity and subglottic pressure in anaesthetized rats: insights from mesencephalic regions

**DOI:** 10.1007/s00424-024-02976-3

**Published:** 2024-06-10

**Authors:** M. González-García, L. Carrillo-Franco, C. Morales-Luque, M. Ponce-Velasco, B. Gago, M. S. Dawid-Milner, M. V. López-González

**Affiliations:** 1 Department of Human Physiology, Faculty of Medicine, University of Málaga, Málaga, Spain; 2 Unit of Neurophysiology of the Autonomic Nervous System (CIMES), University of Málaga, Málaga, Spain; 3 IBIMA Plataforma BIONAND, Málaga, Spain; 4 Department of Cell Biology, University of Málaga, Málaga, Spain

**Keywords:** Dorsolateral periaqueductal gray matter, Subglottic pressure, Nucleus ambiguous, Laryngeal motoneurons, Central cardiorespiratory control, Rat

## Abstract

To assess the possible interactions between the dorsolateral periaqueductal gray matter (dlPAG) and the different domains of the nucleus ambiguus (nA), we have examined the pattern of double-staining c-Fos/FoxP2 protein immunoreactivity (c-Fos-ir/FoxP2-ir) and tyrosine hydroxylase (TH) throughout the rostrocaudal extent of nA in spontaneously breathing anaesthetised male Sprague–Dawley rats during dlPAG electrical stimulation. Activation of the dlPAG elicited a selective increase in c-Fos-ir with an ipsilateral predominance in the somatas of the loose (*p* < 0.05) and compact formation (*p* < 0.01) within the nA and confirmed the expression of FoxP2 bilaterally in all the domains within the nA. A second group of experiments was made to examine the importance of the dlPAG in modulating the laryngeal response evoked after electrical or chemical (glutamate) dlPAG stimulations. Both electrical and chemical stimulations evoked a significant decrease in laryngeal resistance (subglottal pressure) (*p* < 0.001) accompanied with an increase in respiratory rate together with a pressor and tachycardic response. The results of our study contribute to new data on the role of the mesencephalic neuronal circuits in the control mechanisms of subglottic pressure and laryngeal activity.

**Supplementary Information:**

The online version contains supplementary material available at 10.1007/s00424-024-02976-3.

## Introduction

Chemical microstimulation of the mesencephalic periaqueductal gray matter (PAG) has revealed that the different columns within the PAG coordinate different types of responses depending on the stimulus [1, 3, 8, 24], allowing the modulation of an autonomic response dependent on the type of stress and the individual’s subjective perception of a threat or stressful stimulus. For this purpose, the PAG presents many afferences and efferences projections. The most important afferences have their origin in the prefrontal cortex, amygdala and hypothalamus [7, 20, 23, 40, 46, 47]. The PAG, in turn, projects to the pontomedullary cardiorespiratory nuclei involved in cardiorespiratory rhythmogenesis that allows the development of different patterns of cardiorespiratory and motor responses depending on the type of stimulus [8, 9, 21, 26].

Moreover, the PAG assumes a pivotal role in the regulation of vocalisations [18, 42]. Chemical microstimulation within the PAG (lateral or ventrolateral columns) produces vocalisations in cats [44], monkeys [19], birds [36] and humans [17] corresponding with the sound part of human speech. This is facilitated by its connectivity with the laryngeal motor cortex [6, 19] and with the nucleus retroambiguus (nRA) [17, 48]. The nRA is positioned caudal to the pre-Bötzinger complex and contains inspiratory premotor neurons in its rostral section called the rostral ventral respiratory group and expiratory premotor neurons in its caudal section called caudal the ventral respiratory group (cVRG) [21, 29]. These nRA premotor neurons are the perfect target to convert passive breathing into active breathing to generate motor activities that produce changes in abdominal pressure, in addition to modifying the activity of the motor neurons that are located in the nucleus ambiguus (nA) and that control the patency of the pharynx and larynx [4, 12, 16, 43, 45]. In turn, the dorsolateral periaqueductal gray matter (dlPAG) column controls fight/flight or coping/fighting behavioural responses, triggering a defence response that is haemodynamically characterised by hypertension, tachycardia and redistribution of blood flow to the skeletal muscles of the extremities from the abdominal and visceral area. In addition, the response is accompanied by mydriasis, tachypnoea and increased tidal volume. This allows the animal to cope with environmental stress situations that require a rapid cardiorespiratory response [26, 30, 47].

Regarding respiration and vocalisation, it is important to emphasise the need for precise control of the larynx to achieve perfect coordination between both functions. The larynx performs three pivotal functions: respiration, protection (cough and swallowing reflexes) and phonation. Each of these laryngeal functions involves distinctive movements of the vocal folds [27, 34]. The contraction and relaxation of the laryngeal muscles actively modulate the opening and closing of the vocal folds, thereby intricately regulating the egress of air and the subglottic pressure, which denotes the air pressure build-up beneath the vocal folds within the subglottic region of the trachea. Proper synchronisation between subglottic pressure and glottic opening profoundly influences proficient phonation and optimum vocal quality. Vocal fold movements are characterised as adduction and abduction. Therefore, during vocal fold closure, subglottic pressure escalates due to the muscular contraction of respiratory muscles and vocal fold occlusion [17]. The motor neurons that govern the laryngeal musculature primarily reside within the nA of the medulla oblongata [15]. The nA can be anatomically partitioned into three principal domains: the compact formation, housing motor neurons innervating the oesophagus; the semicompact formation, accommodating motor neurons that innervate the pharynx and the cricothyroid muscle, the latter being supplied by the superior laryngeal nerve; and the loose formation, harbouring motor neurons that innervate the remaining laryngeal muscles, excluding the cricothyroid [2, 33].

In previous works, we and others have demonstrated how the parabrachial complex (PBc) and the A5 region (A5) of the pons have a role in the defence response evoked from dlPAG [9, 13, 26] and in modifying the activity of laryngeal motoneurons [22]. In that latter work, the activity of the laryngeal motor neurons of nA and the reflex mechanisms involved in respiratory laryngeal responses were characterised through the technique of the “isolated glottis in situ”. This technique has some advantages, as it separates the lower airways from the larynx, facilitates the evaluation of the changes in neuro-muscular tone and abolishes the influence of the changes of airflow activating laryngeal reflexes [5, 22]. Thus, the PAG seems to modulate the activity of several pontomedullary structures responsible for generating the complete array of laryngeal-respiratory motor patterns imperative for defensive and vocal production [12, 43].

Furthermore, some studies in animal models that communicate vocally and/or learn to speak, such as mice, birds or humans, have shown that the transcription factor FoxP2 (Forkhead box protein P2) is involved in the acquisition of fine motor skills necessary for the production of species-specific vocalisations. These studies show that FoxP2 has a highly conserved role through evolution in the development of language in these species, particularly in mammals [10, 11, 37, 38]. Although FoxP2 protein is expressed in various body tissues during development (pulmonary, nervous, cardiovascular and intestinal), it has a particularly strong presence in brain regions involved in cognitive function, learning, language production and comprehension [31, 39]. A high expression of FoxP2 protein at PAG, PBc and A5 region has also been described [41].

However, the relation between PAG and nA is not well defined. Therefore, the main objective of this work was to characterise the anatomo-functional interactions between mesencephalic and medullary neuronal circuits, especially from the dlPAG region that possibly controls directly or indirectly the activity of nA laryngeal motor neurons in the rat. To this end, we have analysed the pattern of double-staining c-Fos or FoxP2 immunoreactivity (c-Fos-ir, FoxP2-ir) and tyrosine hydroxylase (TH-ir), throughout the rostrocaudal extent of the nA region of anaesthetized male Sprague–Dawley rats during dlPAG electrical stimulation. Subsequently, in the second phase of our study, we have updated the “isolated glottis in situ” technique from previous work and have been able to measure subglottic pressure by stimulating the dlPAG both electrically and chemically. Thus, we were able to verify how dlPAG plays a role in upper airway patency during dlPAG-evoked defence behaviour in anaesthetised animals.

## Material and methods

### Animals and housing

Experiments were conducted on 31 adult male SPF Sprague–Dawley rats weighing between 250 and 350 g, procured from Charles River (Barcelona, Spain). The rats were housed in groups of six per cage, residing in a climate-controlled chamber maintained at a temperature of 22–24 °C and adhering to a 12:12-h light–dark cycle with lights on at 8:00 am. These animals were maintained in the Animal House at the University of Málaga, with unrestricted access to food and water.

### General procedures

The surgical interventions employed in this study were based on the methodologies described in previous publications [9, 25].

All surgical procedures were performed under anaesthesia induced by sodium pentobarbitone (initial dose of 60 mg kg^−1^ i.p., supplemented with 2 mg kg^−1^ i.v. as needed). Cannulation of the femoral artery and vein was carried out for the purpose of arterial blood pressure measurement and drug administration, respectively. To enable the measurement of respiratory flow using a Fleisch pneumotachograph and pleural pressure utilising an air-filled catheter, both the trachea and oesophagus were cannulated. The animals spontaneously breathed a humidified mixture of oxygen-enriched room air. To gauge end-tidal CO_2_ levels during the experiment, a fast response CO_2_ analyser (ADC FM1, The Analytical Development Co., Ltd., Great Amwell, UK) was employed, and values ranged between 3 and 5%. Throughout the experimental procedures, measures were taken to maintain the animals’ body temperature at 37 °C utilising a heated surgical table and a servo-controlled heating pad that monitored rectal temperature, thereby reducing the risk of hypothermia. Anaesthesia levels were continuously monitored during the procedures and throughout the duration of the experiment. This was achieved by assessing any potential alterations in cardiovascular variables under resting conditions and evaluating the absence of a significant withdrawal reflex upon paw pinching. To ensure stability and immobility, the animals’ heads were securely fixed in a stereotaxic frame, with the upper incisor bar positioned 3.3 mm below the interaural line.

### Interaction of dlPAG and nA: c-Fos/FoxP2/TH-ir experiments

In a group of 10 animals, a hole was drilled into the skull to provide access to the right dlPAG. A concentric bipolar electrode (Rhodes Medical Electrodes, SNE-100) was positioned in the right dlPAG. The stereotaxic coordinates to locate the right dlPAG were from − 4.0 mm caudal to bregma, 0.6 lateral to midline and 4.5 to 5 mm depth from the surface of the calota (approaching with an angle of 30°) based on the coordinates provided by the atlas of Paxinos and Watson [35]. The right dlPAG was stimulated once using 1-ms pulses, ranging from 30–50 μA, at a frequency of 100 Hertz for a duration of 5 s, in order to elicit the classical defence response evoked by this region. Following this, guanethidine (10 mg/kg intravenous) was administered to suppress sympathetically mediated cardiovascular responses.

Following this, in one subgroup of animals (*n* = 5), the right dlPAG was stimulated using trains of 1-ms pulses, between 30 and 50 μA, at a frequency of 100 Hertz for 5 s, every 60 s for a period of 1 h. The other subgroup of animals (*n* = 5) did not receive any stimulation and served as the control group.

One hour after the completion of the experiments, the animals were deeply anaesthetized and subjected to perfusion through the ascending aorta using a phosphate-buffered saline (PBS, pH 7.4) solution, followed immediately by a solution of 4% paraformaldehyde in 0.1 M PBS (pH 7.4) to serve as the fixative. The brains were rapidly extracted and subjected to additional fixation by immersion in the same fixative solution for 24 h at 4 °C. Subsequently, the brains were cryoprotected using 30% phosphate-buffered sucrose for several days. The brainstem was then sectioned into 30-μm coronal sections obtained with a freezing microtome (CM 1325, Leica, Wetzlar, Germany). Sampling was performed by selecting every fifth section with a random starting point, and these sections were further processed to assess c-Fos/FoxP2/TH-ir at the level of nA.

#### c-Fos-TH double immunohistochemistry

Sections collected from control and stimulated rats were processed under identical *free-floating* immunohistochemical conditions. The sections were rinsed in 0.1 M PBS (pH 7.4) and pre-treated for 15 min in 3% H_2_O_2_ to eliminate endogenous peroxidase activity. After a second round of washing in PBS saline, sections were incubated overnight at room temperature (RT), using a mouse anti-c-Fos monoclonal primary antibody (1:1000 dilution in PBS containing 0.3% Triton X-100 0.2% Azida 0.02%; Santa Cruz Biotech, ref SC-271243). A biotinylated secondary antibody (1:500 dilution; goat anti-mouse; Vector Laboratories) and ExtrAvidin Peroxidase (1:2000 dilution; Sigma-Aldrich) were used for subsequent incubations, of 1 h at RT each. After washing in PBS (pH 7.4), staining was carried out with 3,3′-diaminobenzidine (DAB; 25 mg in 100 ml Tris–HCl, pH 7.4 and 0.002% H_2_O_2_; Sigma-Aldrich) in the presence of nickel sulphate (1%) to produce a dark reaction product. In order to locate the nA more accurately, TH-ir was only used as a visual reference for helping to locate the ventral medulla. The same sections were then incubated overnight at RT with a polyclonal primary antibody against the tyrosine hydroxylase enzyme (1:5000 dilution; anti-rabbit; Sigma-Aldrich, ref T2928), followed by re-incubation for 1 h at RT in a biotinylated secondary antibody (1:500 dilution; goat anti-rabbit; Vector Laboratories). The TH-ir was revealed only by reacting DAB to produce brown labelling. Sections were dehydrated and coverslipped with DPX mounting medium.

#### FoxP2-TH double immunohistochemistry

This double labelling was performed under the same protocol as for c-Fos-TH-ir but using a sheep anti-FoxP2 primary antibody (1:1000 dilution; RD Systems, ref AF5647) and a biotinylated secondary antibody (1:500 dilution; rabbit anti-sheep; Vector Laboratories).

#### Cell counting and statistical analysis

The rostrocaudal level of each brain section was determined using the Paxinos and Watson rat brain atlas [40]. The cell bodies displaying c-Fos-ir or FoxP2-ir were identified by the presence of a dark nucleus and were quantified bilaterally in the sections containing the nA using a BX61VS optical microscope (Olympus), an Olympus VS software for brightfield image acquisition and QuPath Software. All data were presented as mean ± SEM. To compare the differences between groups, a one-way analysis of variance (ANOVA) was employed for statistical analyses. The significance level was set at *p* < 0.05.

Cell quantification along the nA was executed in accordance with the categorisation into three primary subdivisions (*compact*, *semicompact*, and *loose formation*). To this end, distances from Bregma, as per the Paxinos atlas [35], were employed to guide the selection of slices across the three domains of the nA. Specifically, the criterion for demarcating cells within the *compact region* of the nA spanned from Bregma − 12.00 to − 12.84 mm, the *semicompact region* extended from Bregma − 12.84 to − 13.44 mm and the *loose formation region* encompassed Bregma − 13.44 to − 14.16 mm.

### Interaction of dlPAG and nA: electrical/chemical stimulation and subglottic pressure measurement

In 21 animals, divided into 3 groups, the described general procedures were modified. A double tracheal cannulation was carried out. One cannula was placed upwards in the direction of the glottis for the “glottis isolated in situ” technique. A second cannula was placed downwards in the direction of the carina to measure respiratory flow. That allows us the recording of subglottic pressure and respiratory airflow separately [22]. Subglottic pressure changes are related to changes in laryngeal resistance (glottic opening and closing) because it is the only structure that can influence this variable. In fact, to avoid subglottic pressure changes due to tongue movements, which are possible during PAG stimulation, we fixed the tongue to rule out this variable.

It was of our particular interest to reapply this technique to measure changes in subglottic pressure associated with the cardiorespiratory response evoked from dlPAG stimulation. For this purpose, we have maintained the original design, updating the means of obtaining the subglottic pressure variable thanks to an aneroid transducer (ADInstrument model FE141, ± 0.03 psi) that allows us to pass a stream of humidified warm medical air upwards through the larynx at a constant rate of 30–70 ml/min with a thermal mass digital air flow metre controller (Bronkhorst Hi-Tec F-201CV-AGD-22-V). Thus, at constant air flow, changes in pressure indicate changes in laryngeal resistance. In addition, we have upgraded both the data processing software (LabChartPro) and the signal acquisition hardware (PowerLab 16/30). Thanks to these updates, we have been able to improve the sensitivity and stability of the subglottic pressure signal. In fact, in our study, we have included for statistical analysis only data from animals in which stable recordings were observed before, during and after dlPAG stimulation.

#### dlPAG electrical stimulation and subglottic pressure measurement

In a subset of 7 animals, a burr hole was drilled into the skull of each animal to access the right dlPAG. The right dlPAG was stimulated with 1-ms pulses, 30–50 µA given at 100 Hz for 5 s by positioning a concentric bipolar electrode (SNE-100; Rhodes Medical Electrodes, Summerland, CA, USA).

#### dlPAG chemical stimulation and subglottic pressure measurement

In 14 animals, a burr hole was drilled into the skull of each animal to access the right dlPAG. Microinjections of a solution of PBS (50 nl, pH 7.4 ± 0.1, 5-s duration) (*n* = 7) or glutamate (0.25 M, 50 nl) (*n* = 7), through a stereotaxically positioned single glass micropipette (Hamilton 1.0 µl Model 7001 Knurled Hub (KH) Neuros Syringe), were performed in the same coordinates cited above. Evans blue was used to dissolve all drugs and served to mark microinjection sites. Microinjections of PBS-Evans blue alone were used for control purposes. Microinjection volumes of 50 nl were programmed with a micropump controller (Ultra Micro Pump II, Micro 4; World Precision Instruments, Inc., Sarasota, FL, USA), driving 1-µl microsyringes attached to the micropump. Only one microinjection was delivered to each animal.

Electrical lesions (250 μA DC for 20 s) or Evans blue serve to locate dlPAG electrical stimulation/microinjections sites, respectively. Brains were perfused with 10% formal saline and serially sectioned (50 µm) at the level of the midbrain. The midbrain was counterstained with neutral red.

Different physiological parameters were analysed in each group of experiments under the following protocol:

- Study of laryngeal and cardiorespiratory parameters at rest
- Study of laryngeal and cardiorespiratory changes during dlPAG stimulation (electrical/chemical)

In each animal airflow, pleural pressure (as an index of inspiratory activity), subglottic pressure and arterial pressure were monitored and stored on PC. Measurements were made of instantaneous respiratory frequency, subglottic pressure, mean blood arterial pressure and instantaneous heart rate. In all experiments, baseline values of the parameters were measured immediately prior to dlPAG electrical/chemical stimulation and after (4 min after electrical stimulation and 30 min after chemical stimulation). Changes in mean arterial blood pressure or heart rate were assessed by measuring the peak rise in blood pressure or heart rate observed during the 5-s electrical stimulation of the dlPAG or the maximum cardiovascular response in the chemical stimulation experiments. Stimulus-evoked changes in respiratory parameters were measured as the average response observed during the 5-s electrical stimulation of the dlPAG or the maximum respiratory response in the chemical stimulation experiments. The parameter monitoring and the offline analysis were done using LabChartPro (PowerLab System, ADInstruments®/LabChart Software, version 8.0, Sydney, Australia).

#### Statistical analysis

All data are expressed as mean ± SEM. For statistical comparisons, once the statistical normality (Kolmogorov–Smirnov’s test) and the homoscedasticity (Bartlett’s test) of the data were verified, a paired-sample test was applied to compare the control with the evoked response period for each animal. One-way ANOVA was used to compare different groups of animals. At each time point, *p* < 0.05 was regarded as significant. Only data from animals in which the histology showed that the microelectrodes were positioned within the dlPAG were used for statistical procedures.

## Results

### Interaction of dlPAG and nA: c-Fos/FoxP2/TH-ir experiments

#### Distribution of FoxP2-ir

##### Control animals

In non-stimulated animals, no significant differences were observed regarding the number of FoxP2-ir profiles between ipsilateral and contralateral sides in any of the three principal domains of the nA (Table 1, Fig. 2).

**Table 1:** Relative quantification and distribution of c-Fos and FoxP2 immunoreactive cells (c-Fos-ir and FoxP2-ir) within the nA and nRA in not stimulated animals (control) and dlPAG electrically stimulated animals (stimulated). Both groups are spontaneously breathing rats in which sympathetically mediated cardiovascular changes were abolished with guanethidine. The nA has been divided into its 3 classical domains (loose, semicompact and compact), and ipsilateral and contralateral sides were also analysed

Neuromorphological study					
		Control (*n* = 5)		Stimulated (*n* = 5)	
		Ipsilateral	Contralateral	Ipsilateral	Contralateral
Loose formation	c-Fos-ir cells	29.6 ± 5.1**	18.2 ± 2.7	42.8 ± 5.8***	22.6 ± 4.9
	FoxP2-ir cells	115 ± 18.9	93.4 ± 14.8	115.4 ± 15.5*	88.8 ± 17.6
	Delta c-Fos-ir	11.4 ± 2.4		20.2 ± 2.4^†^	
	Delta FoxP2-irt	21.6 ± 8.1		26.6 ± 2.1	
Semicompact formation	c-Fos-ir cells	23.6 ± 3.8**	14.6 ± 3.3	31 ± 3.3***	16.8 ± 2.4
	FoxP2-ir cells	105 ± 34.8	75.3 ± 26.6	104.5 ± 26.2*	81.5 ± 24.0
	Delta c-Fos-ir	9 ± 1.7		14.2 ± 1.8	
	Delta FoxP2-ir	29.8 ± 12.6		23 ± 5.4	
Compact formation	c-Fos-ir cells	1.6 ± 0.2	1.4 ± 0.2	3.4 ± 0.8*	0.4 ± 0.4
	FoxP2-ir cells	10.8 ± 2.3	4.5 ± 0.9	10 ± 4.7	6.5 ± 3.7
	Delta c-Fos-ir	0.2 ± 0.2		3 ± 0.8^†^	
	Delta FoxP2-ir	6.3 ± 1.8		3.5 ± 1.2	
nRA	c-Fos-ir cells	20.8 ± 2.6	14.4 ± 1.5	26.2 ± 7.3	15.2 ± 5.2
	FoxP2-ir cells	105.3 ± 15.8	75.8 ± 13.1	108 ± 17.8	80.5 ± 13
	Delta c-Fos-ir	6.4 ± 1.6		11 ± 3.1	
	Delta FoxP2-ir	29.5 ± 10.7		27.8 ± 5.8	

Data are expressed as mean ± SEM

^*^p < 0.05, **p < 0.01, and **p < 0.001. Asterisks show differences between ipsilateral and contralateral sides in control or stimulated animals

^†^*p* < 0.05. Daggers show differences between the delta differences between control and stimulated groups

##### Electrically stimulated animals

**Fig. 1:**
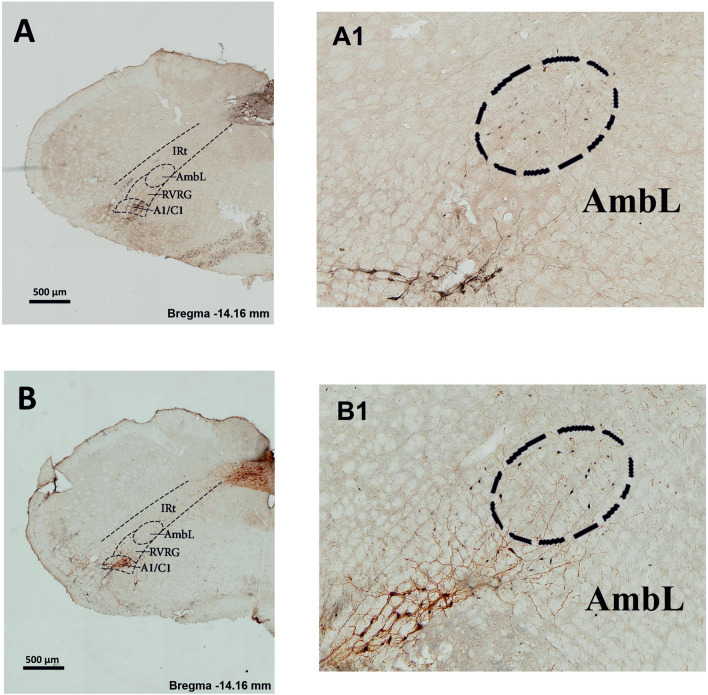
Photomicrographs of coronal sections of the medulla oblongata (stimulated animal), which include the loose formation of the nucleus ambiguus (nA) (bregma 14.16 mm), showing immunoreactivity (ir) for tyrosine hydroxylase (TH) and either FoxP2 (**A**, **A1**) or c-Fos (**B**, **B1**). **A1** Enlarge representative ipsilateral side FOXP2/TH-ir cells; **B1** enlarge representative ipsilateral side c-Fos/TH-ir cells. Abbreviations: C1/A1, adrenaline/noradrenaline cells; RVRG, rostral ventral respiratory group; AmbL, ambiguus nucleus (loose formation); IRt, intermediate reticular nucleus

**Fig. 2:**
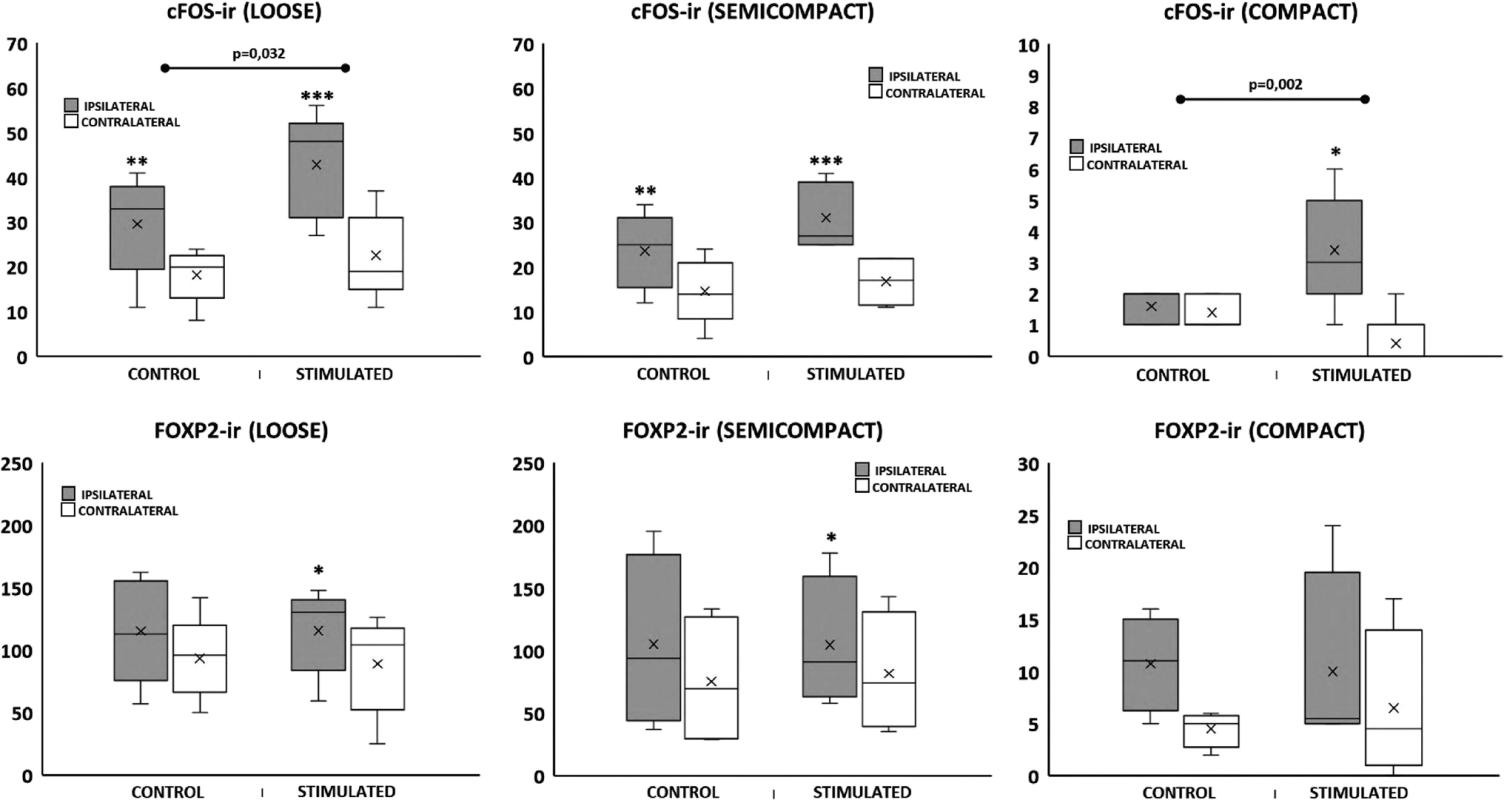
Box-Whisker graphs showing the significant delta change for the number of immunolabelled cells for c-Fos-ir or FoxP2-ir between ipsilateral and contralateral sides, comparing control and dlPAG stimulated (stimulated) animals within the three domains of the nucleus ambiguus (nA). *p < 0.05, **p < 0.01, and ***p < 0.001 ipsilateral (filled box) vs contralateral (empty box)

Both loose formation (*p* < 0.05) and semicompact formation (*p* < 0.05) presented a significant increase in FoxP2-ir profiles on the ipsilateral side compared with the contralateral side in electrically stimulated animals (Table 1, Figs. 1 and 2).

##### Control vs. stimulated animals

No differences were found between control and electrically stimulated animals when comparing the amplitude of the differences between the number and distribution of ipsilateral or contralateral FoxP2-ir neurons within any of the domains in the nA nuclei (Table 1, Figs. 1 and 2).

#### Distribution of c-Fos-ir

##### Control animals

A higher amount of c-Fos-ir profiles were observed in both the loose formation and semicompact formation on the ipsilateral side compared with the contralateral side (*p* < 0.01) in non-stimulated animals (Table 1, Fig. 2).

##### Electrically stimulated animals

A significant ipsilateral predominance for c-Fos-ir staining in all three domains of nA was observed in electrically stimulated animals: loose formation (*p* < 0.001), semicompact formation (*p* < 0.001) and compact formation (*p* < 0.05) (Table 1, Figs. 1 and 2).

##### Control vs. stimulated animals

When comparing the amplitude of the differences of the c-Fos-ir expression in ipsilateral and contralateral sides in control and stimulated groups, a significantly higher increase was observed in both the loose formation (*p* = 0.032) and the compact formation (*p* = 0.002) (Table 1, Figs. 1 and 2).

### Interaction of dlPAG and nA: electrical/chemical stimulation and subglottic pressure measurement

#### dlPAG electrical stimulation and subglottic pressure measurement

In all animals (*n* = 7), the dlPAG electrical stimulation elicited a cardiorespiratory response characterised by tachypnoea (*p* < 0.01), a decrease in laryngeal resistance (subglottal pressure) (*p* < 0.01) and a pressor response (*p* < 0.001) accompanied with tachycardia (*p* < 0.001) (Table 2, Fig. 4).

**Table 2:** Laryngeal and cardiorespiratory parameters measured before electrical or chemical stimulation of dlPAG (rest), during electrical or chemical stimulation (microinjections of PBS or glutamate) within the dlPAG (stimulation) and 4 min after electrical or 30 min after chemical stimulation of dlPAG (recovery)

Neuropharmacological study			
Electrical	Rest	Stimulation	Recovery
dlPAG (*n* = 7)			
RR (rpm)	104 ± 9	145 ± 15**	105 ± 9
TE (s^−1^)	0.343 ± 0.028	0.233 ± 0.021**	0.341 ± 0.025
TI (s^−1^)	0.261 ± 0.021	0.209 ± 0.026*	0.259 ± 0.019
SGP (cmH_2_O)	8.1 ± 1.3	3.3 ± 0.3**	8.1 ± 1.4
BP (mmHg)	106 ± 3	148 ± 6***	104 ± 4
HR (bpm)	408 ± 10	435 ± 10***	410 ± 7
PBS	Rest	Stimulation	Recovery
dlPAG (*n* = 7)			
RR (rpm)	85 ± 7	86 ± 7	85 ± 7
TE (s^−1^)	0.394 ± 0.001	0.385 ± 0.002	0.393 ± 0.001
TI (s^−1^)	0.311 ± 0.001	0.312 ± 0.001	0.312 ± 0.001
SGP (cmH_2_O)	10.4 ± 0.6	10.5 ± 0.6	10.4 ± 0.6
BP (mmHg)	103 ± 3	104 ± 2	103 ± 2
HR (bpm)	394 ± 8	395 ± 7	393 ± 10
Glutamate	Rest	Stimulation	Recovery
dlPAG (*n* = 7)			
RR (rpm)	81 ± 5	111 ± 13***	83 ± 6
TE (s^−1^)	0.474 ± 0.038	0.346 ± 0.037***	0.472 ± 0.035
TI (s^−1^)	0.287 ± 0.009	0.244 ± 0.023*	0.286 ± 0.008
SGP (cmH_2_O)	9.4 ± 0.4	4.6 ± 0.4***	9.8 ± 0.6
BP (mmHg)	105 ± 3	116 ± 4***	107 ± 4
HR (bpm)	391 ± 8	436 ± 8***	393 ± 10

Data are expressed as mean ± SEM

*RR* respiratory rate, *TE* expiratory time, *TI* inspiratory time, *SGP* subglottic pressure, *BP* blood pressure, *HR* heart rate

^*^p < 0.05, **p < 0.01, ***p < 0.001 stimulation vs. rest

#### dlPAG PBS microinjections and subglottic pressure measurement

**Fig. 3:**
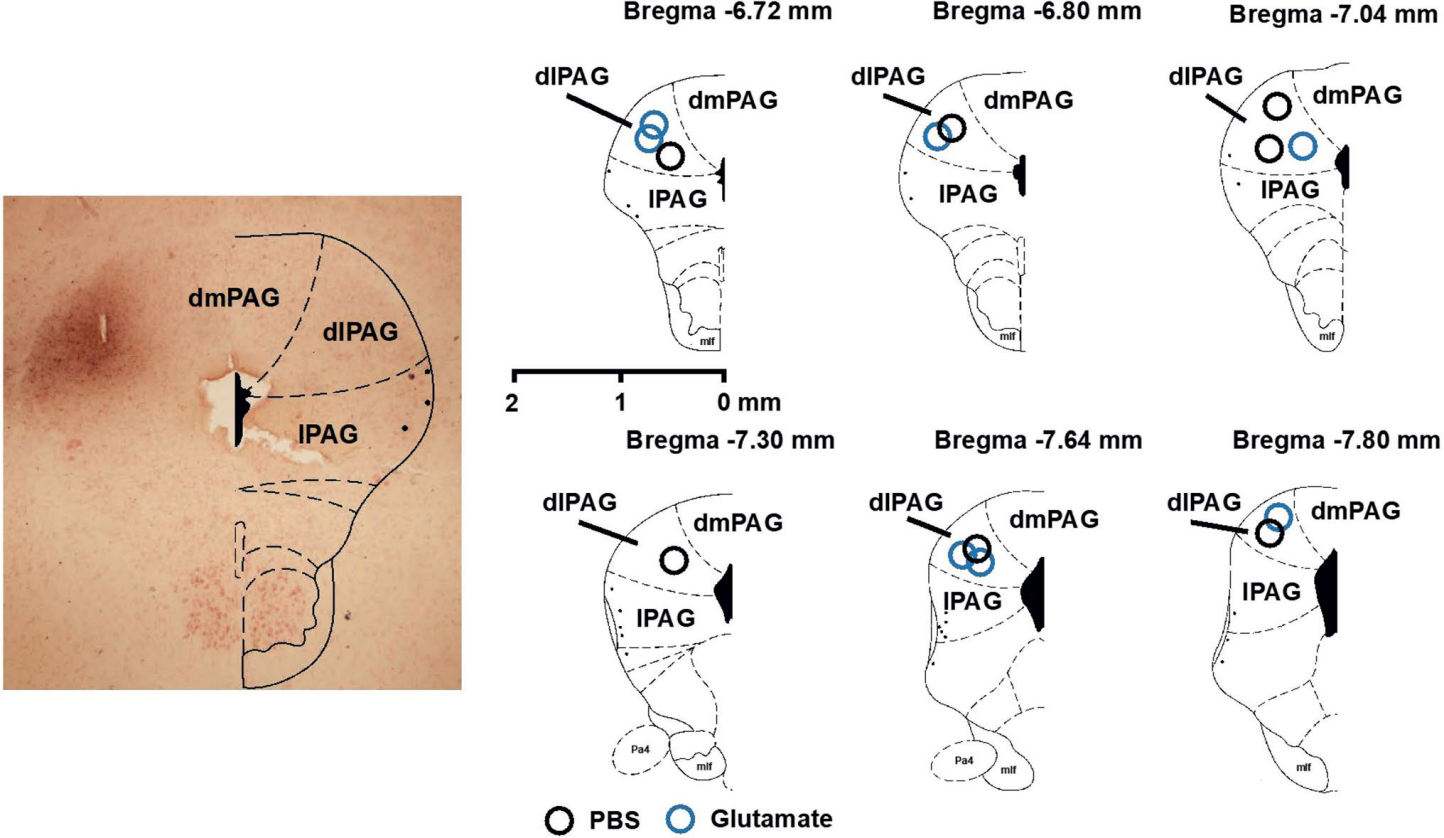
Semi-schematic line drawings of coronal sections from rostral (top left) to caudal (bottom right) areas through the dlPAG, to illustrate the arrangement for chemical microinjections of PBS-Evans Blue (blue circle) or glutamate (black circle) within the dlPAG. Abbreviations: dmPAG, dorso-medial periaqueductal grey; dlPAG, dorso-lateral periaqueductal grey; lPAG, lateral periaqueductal grey; vlPAG, ventro-lateral periaqueductal grey; mlf, medial longitudinal fasciculus; Pa4, paratrochlear nucleus

Microinjections of PBS within the dlPAG (*n* = 7) did not produce changes in any of the resting cardiorespiratory parameters (Table 2, Figs. 3 and 5).

#### dlPAG glutamate microinjections and subglottic pressure measurement

**Fig. 4:**
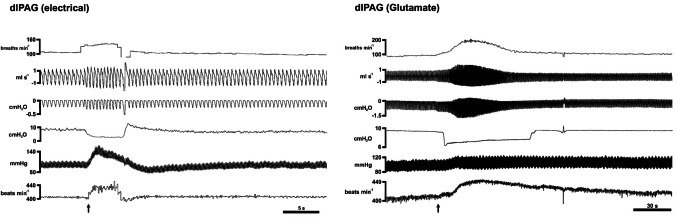
Instantaneous respiratory rate (upper trace, breaths min^−1^), respiratory flow (ml/s), pleural pressure (cm H_2_O), subglottic pressure (cmH_2_O), blood pressure (mmHg) and instantaneous heart rate (beats min^−1^) in a spontaneously breathing rat showing the laryngeal and cardiorespiratory response evoked to dlPAG electrical stimulation (left) and dlPAG glutamate stimulation (right). Black arrow shows the onset of the dlPAG stimulation

**Fig. 5:**
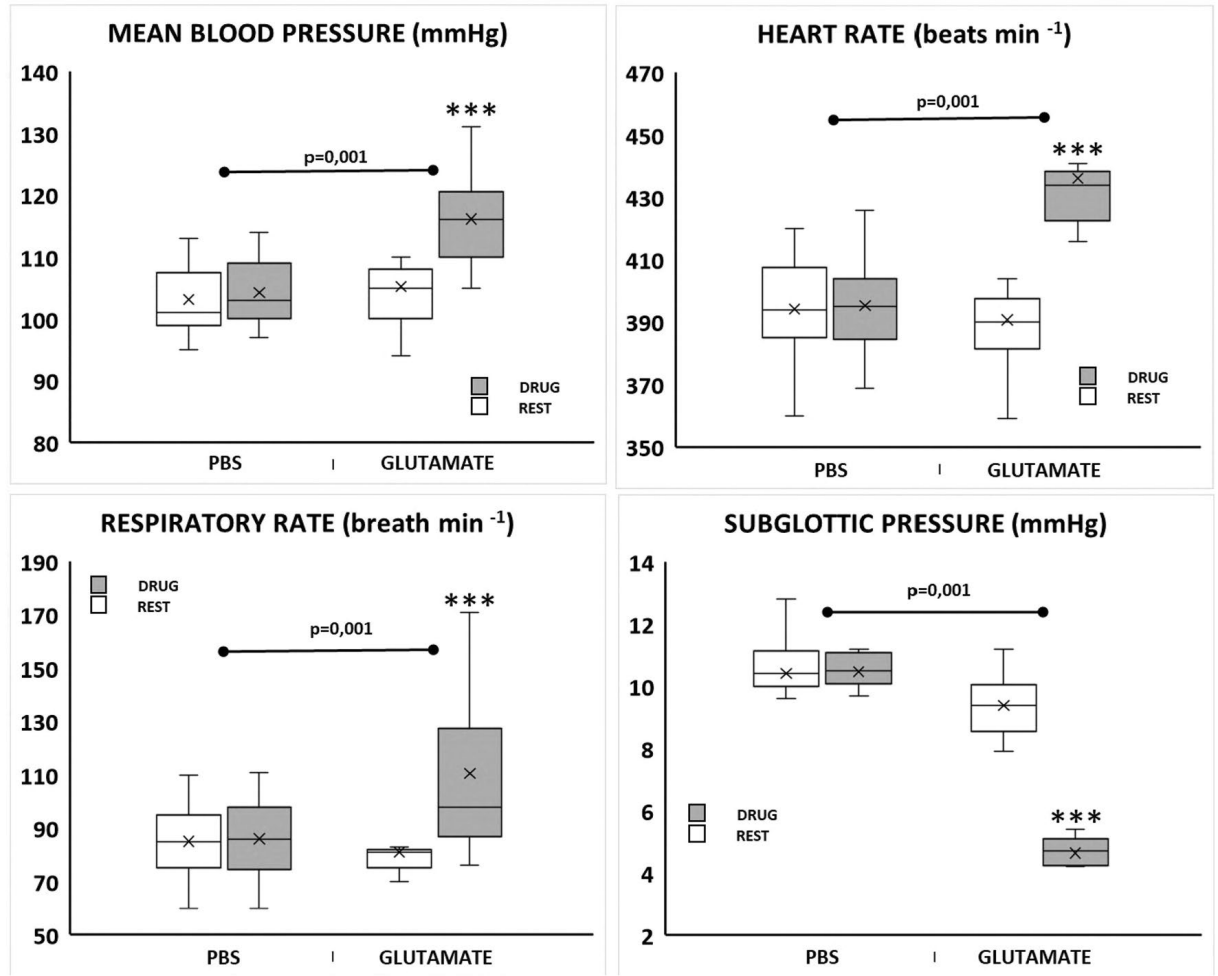
Box-Whisker graphs showing the significant delta change for laryngeal and cardiorespiratory responses (mean blood pressure, heart rate, respiratory rate, and subglottic pressure) between rest (empty box) and stimulation (filled box) states comparing PBS-Evans blue or glutamate microinjections within the dlPAG. ***p < 0.001 stimulation vs. rest

Glutamate microinjections within the dlPAG (*n* = 7) evoked a decrease in laryngeal resistance (subglottal pressure) (*p* < 0.001) accompanied with an inspiratory facilitatory response consisting of an increase in respiratory rate (*p* < 0.001), together with a pressor (*p* < 0.001) and tachycardic response (*p* < 0.001) (Table 2, Figs. 3, 4, and 5).

#### dlPAG PBS vs. glutamate microinjections and subglottic pressure measurement

When comparing the amplitude of the differences in the laryngeal and cardiorespiratory responses between PBS and glutamate microinjected animals, higher values in delta heart rate (0.29 ± 2.5 to 45.57 ± 3.9; *p* = 0.001), delta blood pressure (0.00 ± 1.0 to 10.57 ± 0.82; *p* = 0.001), delta subglottic pressure (0.05 ± 0.05 to − 4.60 ± 0.24; *p* = 0.001) and delta respiratory rate (1 ± 0.69 to 29.57 ± 8.7; *p* = 0.001) were observed in stimulated animals (Fig. 5).

## Discussion

The results of our study demonstrated, using neuromorphological and electroneurophysiological techniques, the existence of functional interactions between the dlPAG, a mesencephalic region known for its involvement in cardiorespiratory regulation, and the nA, a medullary region containing the majority of the motor neurons that govern the laryngeal musculature. Firstly, we demonstrated that electrical stimulation of dlPAG induces a significant increase in c-Fos-ir expression in the loose formation and compact formation domains of the nA. Secondly, we show that the loose formation and semicompact formation regions are the nA subdivisions with the highest expression of FoxP2-ir, while the compact region shows the lowest expression. No significant changes in FoxP2 expression occur after dlPAG stimulation in either region. Finally, using the “isolated glottis in situ” technique together with classical electrophysiological techniques, we demonstrate for the first time that dlPAG is involved in the control of laryngeal resistance.

All these results suggest that, in our experimental conditions, the dlPAG modulates, directly or indirectly, the activity of neurons located within the loose formation of the nA, thereby influencing the striated laryngeal muscles of the upper airway, during the concomitant cardiorespiratory changes evoked by dlPAG electrical or chemical stimulation.

### Interaction of dlPAG and nA: c-Fos and FoxP2 expression

The neuronal cell bodies responsible for innervating the intrinsic muscles of the larynx are situated within nA. This cell column comprises neurons oriented in a rostrocaudal direction and is positioned in the ventrolateral region of the medulla oblongata. Its spatial extent ranges from the motor nucleus of the facial nerve to at least the level of the pyramidal decussation [2]. We could divide the nA into three main parts or domains: the compact formation, with motor neurons innervating the oesophagus; the semicompact formation, with motor neurons innervating the pharynx and the cricothyroid muscle of the larynx, i.e. that which is innervated by the superior laryngeal nerve; and the loose formation, with motor neurons innervating the laryngeal muscles except the cricothyroid. It is therefore accepted that laryngeal neurons are located in the caudal part of the nA (semicompact and loose formation). Thus, the nA, in addition to innervating the laryngeal muscles, also provides motor innervation to the oesophagus and pharynx [14, 33, 49].

In the present study, guanethidine, a sympatholytic agent, was administered to prevent secondary c-Fos expression resulting from alterations in arterial blood pressure. Thus, despite the blockage of the cardiovascular changes, the nA presents a significant increase of ipsilateral c-Fos-ir within the three principal domains of the nA after electrical stimulation of the dlPAG. When comparing the differences between stimulated and control animals in the increase of c-Fos-ir between the ipsilateral and contralateral sides, only the loose formation and compact formation present significant changes in their activity.

Therefore, both populations of neurons within the nA seem likely to be activated directly or indirectly from the dlPAG and not secondarily to blood pressure changes evoked from the dlPAG during electrical stimulation. Furthermore, the analysis of c-Fos-ir within the nRA, indicates that the dlPAG is not affecting the activity of these nRA neurons. Thus, our data suggest that the dlPAG may not play a significant role in the vocalisations emitted during stressful or dangerous situations. Moreover, the changes in c-Fos expression in these regions may be due to the need to maintain a tachypnoeic response developed in the classical defence response vehiculated by this mesencephalic region.

When examining the data of FoxP2 nuclei, we confirmed a high level of expression at the level of semicompact formation (controls the vocal fold tension) and loose formation (regulates the subglottic pressure during vibration) [28], giving a role to the neurons of these domains in the production of laryngeal-respiratory motor patterns necessary for the correct production of species-specific vocalisations. It should be noticed that FoxP2 expression levels were not modified by electrical stimulation of the dlPAG. Regarding these quantitative data from FoxP2-ir, it should be noted that they are in line with previous studies in which numerical approximations were made within these domains [33], confirming that the expression of this transcription factor is a characteristic rather than an expression of cellular activity.

### Interaction of dlPAG and nA: electrical/chemical stimulation experiments and subglottic pressure

In our conditions, electrical stimulation was used primarily to locate dlPAG and to study laryngeal patency through the measure of the subglottal pressure. In a second group of animals, we have developed chemical microstimulation with glutamate microinjections within dlPAG. The results obtained with electrical stimulation are difficult to interpret in isolation. However, if we consider both, these results and the results obtained with chemical stimulation, the effects suggested that the elicited laryngeal and cardiorespiratory responses can be attributed to the activation of cell bodies located within the dlPAG, but not to the activation of axons of passage. Both the chemical and electrical stimulation of the dlPAG produced a sympathoexcitatory response characterised by hypertension, tachycardia and tachypnoea. All these changes were accompanied with a reduction in subglottal pressure. The magnitude of the changes in subglottal pressure and cardiorespiratory variables were similar, except for the arterial pressure, using both methods of stimulation.

The decrease in subglottic pressure obtained in our experimental conditions suggests that the dlPAG, through direct and indirect connections with pontine (PBc/KF, A5) and medullary respiratory nuclei (nRA, nA), decreases laryngeal resistance. We can hypothesise that central respiratory mechanisms are activated during tachypnoea evoked by the defence response. This may override any increase in laryngeal adductor activity that would be expected if the animal intended to vocalise, resulting in a decrease in laryngeal resistance during tachypnoea. In a recent publication [32], it was described how the PAG could facilitate inspiration by projecting to the pre-Bötzinger complex and the nRA, respectively.

Thus, in conclusion, the dlPAG has a role in modifying the activity of laryngeal motoneurons located within the nA and, accordingly, the striated laryngeal muscles of the upper airway during the defence response. Future works are needed to dilucidate if dlPAG could modify the increase of the activity of laryngeal inspiratory neurons and reduce the activity of expiratory ones to facilitate the tachypnoeic evoked response during the defence response evoked from dlPAG stimulation.

### Supplementary Information

Below is the link to the electronic supplementary material.

From top to bottom: subglottic pressure (cmH_2_O), respiratory flow (ml/s) and pleural pressure (cm H_2_O) in a spontaneously breathing rat showing the respiratory modulatory effect over the laryngeal response evoked to dlPAG electrical stimulation. (PDF 1985 KB)

## Data Availability

No datasets were generated or analysed during the current study.
