# Supplementary figures and images for: Uncovering the neural control of laryngeal activity and subglottic pressure in anaesthetized rats: insights from mesencephalic regions
Source: Pflugers Arch. 2024 Jun 10;476(8):1235–47. doi: 10.1007/s00424-024-02976-3 (PMC11271367; doi:10.1007/s00424-024-02976-3)

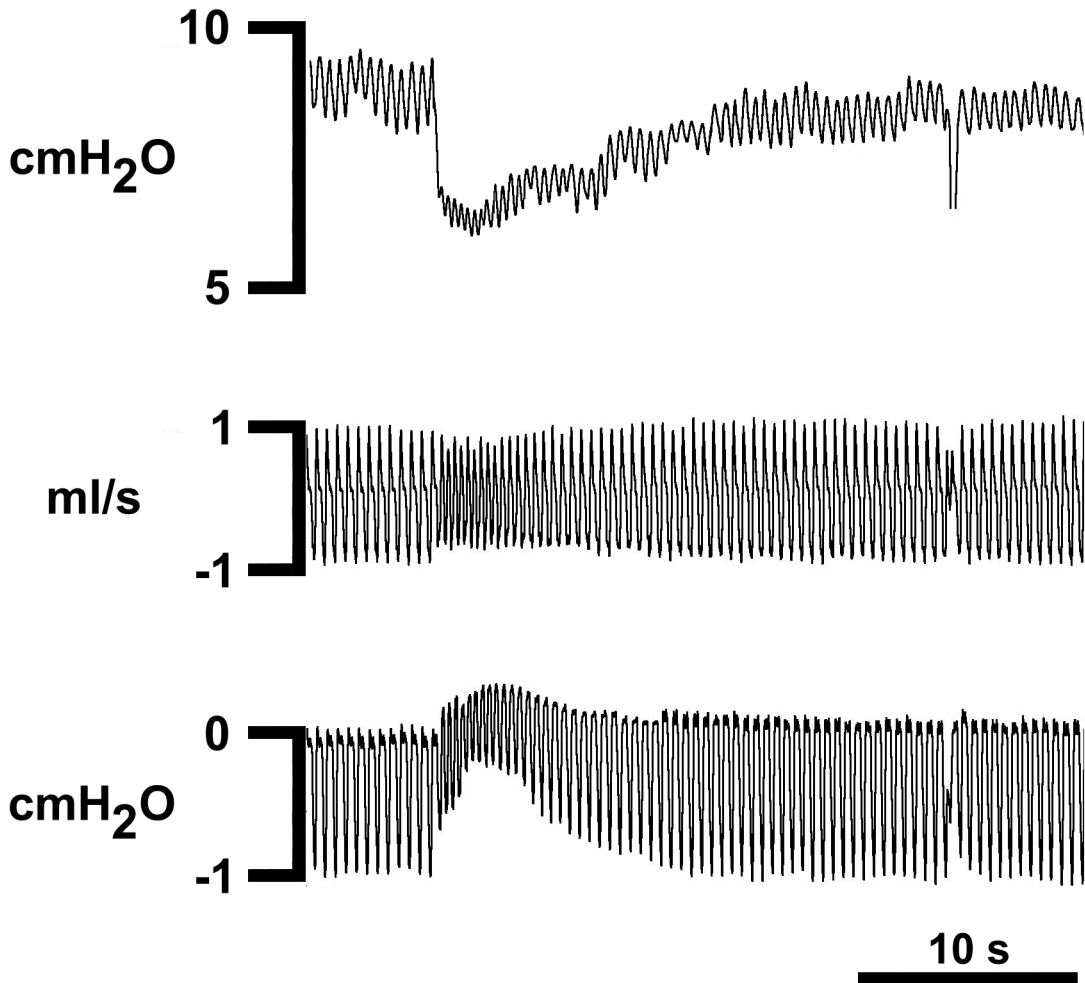

Supplement: Supplementary file 1 — From top to bottom: subglottic pressure (cmH2O), respiratory flow (ml/s) and pleural pressure (cm H2O) in a spontaneously breathing rat showing the respiratory modulatory effect over the laryngeal response evoked to dlPAG electrical stimulation. (PDF 1985 KB) [file 424_2024_2976_MOESM1_ESM.pdf]
